# The Mechanisms of Cadmium Toxicity in Living Organisms

**DOI:** 10.3390/toxics12120875

**Published:** 2024-11-30

**Authors:** Slavena Davidova, Viktor Milushev, Galina Satchanska

**Affiliations:** 1 UPIZ Educational and Research Laboratory of Biology-MF-NBU, New Bulgarian University, 1618 Sofia, Bulgaria; stdavidova@nbu.bg (S.D.); f113742@students.nbu.bg (V.M.); 2 Department of Natural Sciences, New Bulgarian University, Montevideo Blvd., 1618 Sofia, Bulgaria

**Keywords:** Cd pollution, Cd toxicity, bacteria, plants, animals, human, molecular mechanisms

## Abstract

Cadmium (Cd) is a toxic metal primarily found as a by-product of zinc production. Cd was a proven carcinogen, and exposure to this metal has been linked to various adverse health effects, which were first reported in the mid-19th century and thoroughly investigated by the 20th century. The toxicokinetics and dynamics of Cd reveal its propensity for long biological retention and predominant storage in soft tissues. Until the 1950s, Cd pollution was caused by industrial activities, whereas nowadays, the main source is phosphate fertilizers, which strongly contaminate soil and water and affect human health and ecosystems. Cd enters the human body mainly through ingestion and inhalation, with food and tobacco smoke being the primary sources. It accumulates in various organs, particularly the kidney and liver, and is known to cause severe health problems, including renal dysfunction, bone diseases, cardiovascular problems, and many others. On a cellular level, Cd disrupts numerous biological processes, inducing oxidative stress generation and DNA damage. This comprehensive review explores Cd pollution, accumulation, distribution, and biological impacts on bacteria, fungi, edible mushrooms, plants, animals, and humans on a molecular level. Molecular aspects of carcinogenesis, apoptosis, autophagy, specific gene expression, stress protein synthesis, and ROS formation caused by Cd were discussed as well. This paper also summarizes how Cd is removed from contaminated environments and the human body.

## 1. Introduction

Cadmium (Cd, atomic number: 48, atomic mass: 112.4, period number: 5, Group 12, electron configuration: 4d^10^5s^2^, isotopes: 7) was discovered in 1817 by the German chemist Friedrich Stromeyer. This chemical element was named after one of the first heroes in Greek mythology, Cadmus, the legendary founder of Thebes, known for his immense strength before Heracles. The Latin word cadmia and the Greek word καδμεία, which was once used to refer to the standard zinc (Zn) ore calamine, are the sources of the name [1]. Cd was not known to be a separate element until 1817, as in nature, Cd is always found in the presence of zinc. Cd and zinc are chemically and physically similar. Cd is a by-product of zinc and lead production. The Cd/Zn ratio is 0.5% in nonferrous ores, with the actual recovery of cadmium metal estimated at 50–65% of that present in raw ore [2]. Cd is unique among metals due to its diverse toxic effects, long biological half-life, low rate of excretion, and predominant storage in soft tissues rather than bone [3]. In Table 1 are described some of Cd’s physical and chemical properties.

Cd was initially found to have negative health consequences in 1858. Those who used polishing agents containing Cd suffered from respiratory and gastrointestinal problems. The earliest toxicological experiments were conducted in 1919. In humans, emphysema and proteinuria were first documented in the 1940s in workers exposed to Cd dust [3]. Following World War II, a bone ailment known as itai-itai disease—a type of Cd-induced renal osteomalacia—was reported in Japan, causing fractures and excruciating agony [3,4]. The toxicokinetics and toxicodynamics of Cd were then discussed, along with how it binds to the metallothionein protein [5]. In the 1970s, health advisories regarding the dangers of Cd pollution were distributed globally [6]. The World Health Organization’s International Program on Chemical Safety recognized renal failure as a critical consequence of Cd and offered a rough quantitative evaluation. In the 1990s and 2000s, various epidemiological studies found adverse health impacts in demographic groups in Japan, China, Europe, and the United States, sometimes with minimal ambient Cd exposure. The early discovery of metallothionein’s important role in Cd toxicology laid the groundwork for recent studies that used biomarkers of susceptibility to the development of Cd-related renal dysfunction, such as metallothionein gene expression in peripheral lymphocytes and metallothionein autoantibodies in blood plasma [7].

## 2. The Origin of Cd Pollution

**Figure 1:**
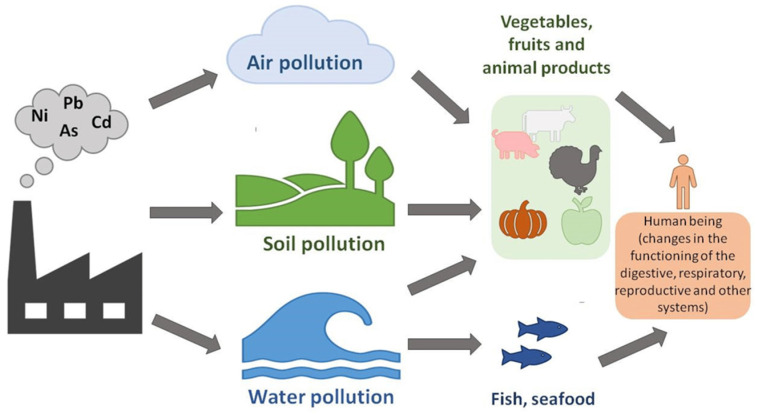
Distribution of heavy metals in the environment [17].

Cd is an environmental contaminant classified eighth on the Top 20 Hazardous Substances Priority List because of its high toxicity and sluggish metabolism [8]. The primary source of Cd is stack dust, produced during zinc purification by distillation and deposited in all fractions [9]. Plants can absorb Cd directly from the soil [10]. Phosphate fertilizers and atmospheric deposition have been the primary sources of Cd intake into soils. Phosphorite and apatite rocks mainly used in the production of phosphate fertilizers contain Cd and several other heavy metals [11]. The amount of Cd accumulated in soil due to environmental contamination depends on the magnitude of emissions, transit, and retention. The fate of heavy metal contaminants in soil is mainly determined by the balance of sorption, leaching, and plant uptake. Soil variables like pH, redox state, organic matter, clay, hydrous oxides, and free carbonates significantly impact these processes. Metal destiny varies significantly amongst soil types, including forest and heavily developed agricultural land [12]. Global production and use of Cd are significant; for instance, Cd pigments consumption surpasses 2500 tons annually [13]. For thousands of years, it has also been used as a pigment due to its ability to produce almost all the rainbow colors: brilliant yellow, orange, and red, interacting with other chemical elements. Thus, a bright orange color is made when the bright yellow pigment cadmium sulfide (CdS) is mixed with cadmium selenite (CdSe). Cadmium sulphoselenide (Cd_2_SSe) creates a pigment called cadmium red. The pigments mentioned above are still used nowadays as plastic colorants. In principle, Cd is part of various chemical compounds as a divalent cation [14]. Human activities associated with Cd emissions include industrial production, crop farming, animal breeding, aquaculture, and wastewater treatment [15]. Low amounts of Cd are naturally found in the lithosphere (0.15 mg/kg in the Earth’s crust and 1.1 × 10^−4^ mg/L in seawater) [16]. Still, various industrial processes, including mining and smelting, have increased the element’s availability in the environment and increased human exposure to it. Thousands of tons of garbage polluted with Cd are dumped into the environment globally each year [16]. In Figure 1 is presented the distribution of heavy metals in the environment.

The Jinding lead-zinc mine is currently the largest lead-zinc deposit proven in China, one of only a few with over 10-million-ton reserves worldwide. Its mining area is 6.8 km^2^, with more than 80% open-pit mining. The large-scale mining of the Jinding Pb-Zn deposit began in the 1980s, causing significant environmental problems [18]. Descriptive statistics revealed that the 1.1% As, 7.3% Cd, 0.3% Pb, and 0.2% Hg contents exceeded China’s Soil Environmental Quality Management Standard (GB 15618-2018, in Chinese) [19]. Furthermore, 32.8% of As, 74.4% of Cd, 89.2% of Pb, 45.0% of Cr, and 13.7% of Hg concentrations in soil samples exceeded the background soil concentrations of heavy metals in this location, with Cd and Pb having the highest levels, which were 11.64- and 21.47-fold the background values. On the other hand, compared to regular farming areas, industrial and mining enterprises, sewage irrigation, and urban samples from those areas had considerably greater concentrations of As, Cd, Pb, Cr, or Hg in their soil [20]. Surface waters, especially the lakes are a significant global source of freshwater, and Cd pollution of water bodies is becoming more of an issue as industry, agriculture, and other human activities advance [21]. Cd pollution in lakes poses a significant risk to water quality, drinking water supplies, the food chain, and freshwater ecosystems. Sediments have been observed to act as both a sink and a source of Cd in water bodies. Yearlong monitoring in Meiliang Bay (northern part of Taihu Lake, National Wetland Park, eastern China) revealed that the mobility of Cd in sediments varied widely and significantly impacted the Cd pollution level in the overlying water [8]. Tourism has been shown to contribute to Cd pollution (primarily through hotel wastewater and increased traffic) and vice versa. Cd pollution of beaches, coastal waterways, food, urban parks, and other areas poses risks to tourists and increases human exposure to this poisonous metal [22].

### 2.1. Natural Sources of Cd

#### 2.1.1. Cd in Soil Water and Groundwater

**Figure 2:**
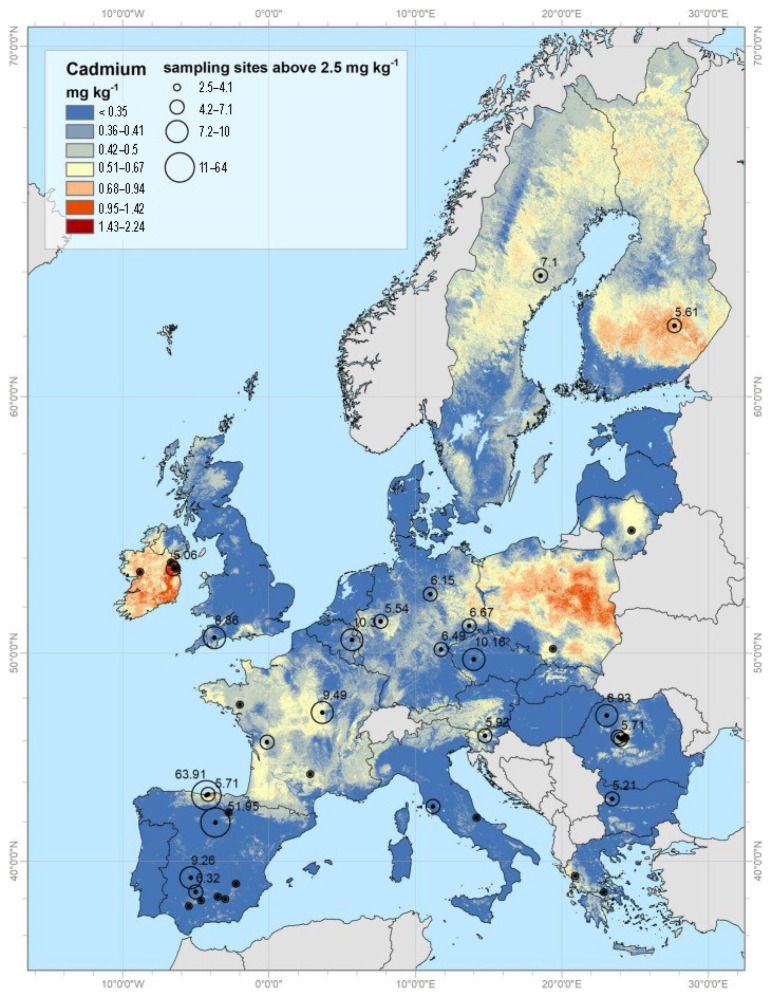
Cd concentration for 2024 in Europe with the outliers overlaid as black circles. The dimension of the circle indicates the concentration for the given outlier; concentrations above 5 mg/kg^−1^ are also indicated by their numerical value [25]. As seen from the figure, the highest Cd concentration was registered in northern Spain.

Generally, Cd concentration in the earth is around 0.1–0.5 ppm, and this metal mainly accumulates in sedimentary rocks. Natural activities such as erosion, weathering of rocks, volcano eruptions, and wildfires release large amounts of Cd into soils and rivers and, respectively, seas and oceans. Phosphorites and marine phosphates contain high amounts of Cd, as much as 500 ppm. Maximum permissible Cd concentrations are 5 μg/g in soil and water and 1 μg/L in groundwater [21]. Large quantities of Cd (about 15,000 mt (metric tons) are transported into rivers by erosion and weathering of rock materials. Volcanic eruptions release about 820 metric tons of Cd, while forest fires release up to 70 metric tons [23]. Detailed information on Cd distribution in European top soils is available on the European Commission’s official webpage: https://esdac.jrc.ec.europa.eu/search/node/cadmium [24] (accessed on 20 November 2024). As seen from Figure 2, out of the total, 72.6% of the samples have Cd values < 0.07 mg kg^−1^, 21.6% in the range 0.07–1 mg kg^−1^, and the remaining 5.5% higher than the threshold of 1 mg kg^−1^, which is generally considered the limit for risk assessment [25].

Data about all heavy metal concentrations are permanently deposited at the European Soil Data Centre (ESDAC, https://esdac.jrc.ec.europa.eu/, accessed on 28 October 2024) EU, European Commission, Joint Research Centre. Other data on the topic can be found in the Agency for Toxic Substances and Disease Registry (ATSDR), EFSA CONTAM Panel reports, and the Organisation for Economic Co-operation and Development (OECD).

The quality of surface water in Europe is regulated by Directive 2000/60/EC [26]. There are 100,000 surface water bodies in Europe, but only 40% are in good condition. As Kubier et al. [21] highlighted, WHO Guidelines for Drinking Water Quality recommend a value of up to 3 μg/L.

Groundwater in Pakistan has typical Cd contents of 10 μg/L from Jurassic sulfide-bearing sedimentary rocks. In contrast, in Germany, groundwater concentrations of Cd range from 0.11 μg/L in loess aquifers beneath arable land to 2.7 μg/L in sandy aquifers beneath forested regions [21].

A comparison of crucial aquifer systems reveals a link between rock type, groundwater environment, and Cd contents. Cd’s 90th percentile background levels in groundwater varied from less than 0.1 μg/L in Paleozoic, Triassic, and Jurassic aquifers to more than 1 μg/L in Rotliegend, Cretaceous, and Cenozoic aquifers. Aside from calcium carbonate Cretaceous aquifers, limestone-dominated aquifer basins exhibited low Cd levels in groundwater. Most of them are in alkaline aquifer systems [21]. Cd concentrations above 1 μg/L were found in groundwater in sandstone aquifers and unconsolidated sand and gravel aquifer systems in the western United States. However, in most samples collected from 3124 wells in the US, Cd was below 1 μg/L.

Groundwater, but from a glacial aquifer system in the United States, had Cd values ranging from 0.018 μg/L to 1.0 μg/L. However, 84% of groundwater samples (N = 847) were below the detection limit. A survey of groundwater near garbage sites in the United States revealed Cd values of up to 6000 μg/L. Municipal solid waste dumps in the European Union can produce leachates with Cd values up to 2700 μg/L. As a result, Cd concentrations that exceed the natural background can be caused by both natural and anthropogenic mechanisms [21].

#### 2.1.2. Air

Natural Cd emissions come mainly from rock weathering, airborne soil particles from deserts, sea spray, forest fires, biogenic material, volcanoes, and hydrothermal vents. In Europe, the leading standard regulating the Cd concentration in ambient air is the Directive 2008/50/EC (https://ec.europa.eu/environment/air/quality/directive.htm, accessed on 28 October 2024) [27]. According to this directive, the maximum permission level is 5 ng/m^3^, which has been valid since 2013 [27]. In the USA, this restriction is even more substantial, and the regulatory bodies such as NAAQS (National Ambient Air Quality Standards), USEPA (United States Environmental Protection Energy Agency), and NIOSH (National Institute of Occupational Safety and Health) limited Cd in the air to 200 pg/m^3^. It is known that PM_2.5_ can transport heavy metals such as Cd, Hg, Pb, Cr, and Mn. In research conducted in Beijing, China, the immediate impacts of PM_2.5_ exposure on blood Cd levels were studied. The findings revealed that the average blood Cd concentration was 0.64 μg/L. A significant correlation was observed between PM_2.5_ exposure and blood Cd level (*p* < 0.05), as authors reported [28].

Soil particles are the most common source of natural emissions into the atmosphere, followed by forest and bushfires, sea salt, volcanic emissions, and meteoric dust [29]. Wildfires increase Cd concentrations in soils and ashes. Long-term behavior analysis revealed decreasing Cd concentrations in the solid phase, as rainfall and pH decrease with time following fire, resulting in desorption and mobility of Cd and other heavy metals [21]. Wildfires in California, for example, raised the average Cd concentration in the runoff by more than two orders of magnitude. Cd concentrations in biomass ash can reach 30 mg/kg, providing an additional method for increasing Cd concentrations in soil because such ash is commonly used as fertilizer. In the short term, the bioavailable pool of Cd remains low due to an ash-induced pH increase, but as pH rises due to rainfall, Cd bioavailability increases [21].

### 2.2. Anthropogenic Cd Sources

Anthropogenic Cd inputs into soil, groundwater, and atmosphere come from mining—lead, zinc smelters [30], uranium mill tailings [31,32,33] as reported by Satchanska et al., nonferrous metal manufacturing, fossil fuel combustion, phosphate fertilizer and pesticide manufacturing, iron, steel, cement production, road dust, plastics production, wildfires, and municipal and sewage sludge incineration. Environmental pollution with Cd stems from its widespread use in the production of alloys and batteries, as a pigment in plastics, paints, and ceramics, and corrosion-resistant coatings of metal products. This heavy metal is primarily found and abundant in lead, copper, and zinc ores. Over the past 50 years, anthropogenic Cd emissions have fallen by more than 90% [34]. Like with uranium (U), using phosphate fertilizers with Cd as an impurity is a common cause of high Cd concentrations in soil and groundwater. This Cd addition pathway to groundwater was explored in the United States, Canada, Britain, Norway, Sweden, Finland, Denmark, Germany, Australia, and New Zealand. The findings indicate that P fertilizer application alters soil chemistry. Furthermore, Cd can enter the food chain and be hazardous to living organisms. Cd sources can be both local and diffuse [30].

Local sources such as mines, industrial sites, and abandoned mining deposits cause increased Cd concentrations, albeit generally on a limited spatial scale [35]. Atmospheric emissions, wastewater reuse, and agricultural operations can all act as diffuse sources, resulting in the widespread distribution of Cd in the environment [36].

The screens of mobile phones from different generations showed a significant decrease in the quantities of Cd (from 1.0 μg/g to undetectable levels) and of Pb (from 35.0 μg/g to 2.0 μg/g) from feature phones to smartphones [37]. Cd is also commonly present in children’s toys. As reported by Igweze et al. [38], cheap toys purchased from Port Harcourt, Nigeria, stores were determined to contain three toxic metals (Pb, Cd, and As). The present heavy metals in all the toys were below the limits set by the EU [38].

Heavy metals, including arsenic, cadmium, chromium, and lead, are present in goods made from leathers, synthetic leathers used in shoe production, and textiles. Bielak et al. [39] found that children’s footwear made from sheepskins contained As, Ba, Cd, Cr, Hg, Pb, Sb, and Se. In 2006, leathers used for insoles, shoe uppers, and clothing underwent testing in Turkey for Co, Cr, Cu, Pb, Ni, and Zn due to their close contact with the body. In Bielak et al.’s paper, it is described that in 2015, various types of fibers used in the textile industry in Turkey, such as cotton, acrylic, polyester, nylon, viscose, and polypropylene, were examined for the presence of Al, Cd, Co, Cr, Cu, Fe, Mn, Ni, Pb, Tl, and Zn. Additionally, in Italy in 2009, Cr was identified in a wool top [39].

The utilization of cadmium for creating affordable jewelry has recently become of concern. Kern et al. conducted a study in 2020 and found that seven out of the nine undamaged jewelry items tested released cadmium in amounts that surpassed the recommended maximum of 18 μg. They concluded that the undamaged items had a maximum extractable cadmium amount of 6230 μg, nearly three hundred times greater than the 18 μg limit [40].

Nickel-cadmium batteries are the most common source of Cd in dump sites with municipal solid waste worldwide [41]. In European municipal solid wastes, Cd levels range from 0.3 to 12 mg/kg. Pigments, coatings and platings, PVC stabilizers, and alloys are also Cd-containing items [21].

## 3. Cd Toxicity on Living Organisms

Heavy metals, respectively Cd, exist in soils in two forms—immobilized (organically bound) and mobile (in the soil solution). The immobilized ones are considered nonbioavailable, while the mobile is considered bioavailable. The access of heavy metals to organisms is also affected by different soil characteristics such as pH, oxido-reductive balance, clay, iron oxide, and organic matter content. It is well known that the presence of Cd and other heavy metals in the environment influences the growth and survival of microorganisms and also shifts the microbial community in these habitats, resulting in poorer soil fertility [29,41]. As a rule, Gram-positive bacteria are more sensitive than Gram-negative bacteria [42].

### 3.1. Cd Accumulation and Toxicity on Bacteria

Several studies on the interactions of Cd with microorganisms have been reported [43]. In some bacteria, such as *Ralstonia metallidurans* CH34, isolated near a zinc production plant in Belgium, a gene called the *czc*-gene, responsible for the induction of resistance to Cd, was found [44]. Heavy-metal resistance genes were found to be localized outside this microorganism’s bacterial chromosome in two small (3–5 Kb) self-replicating circular DNA molecules called plasmids pMOL28 and pMOL30. Overview results on bioremediation of Cd by the Gram(−) bacterium *Pseudomonas aeruginosa* are published by Chellaiah [43].

#### 3.1.1. Resistance Mechanisms of Bacteria

Bacteria inhabiting high-metal environments evolved several mechanisms of resistance, fighting for survival. Usually, Cd(II) forms stable toxic complexes in the cell. The concentration of Cd ions in the cell is regulated by cellular homeostasis through these specific resistance mechanisms [45].

Resistance mechanisms of bacteria function as energy-dependent effluxes of metal ions that export them out of the bacterial cell. In most cases, the resistance is carried out by plasmids that contain genes encoding resistance to Cd [37]. Besides Cd^2+^, the plasmid-mediated resistance system removes other metal ions such as Ag^+^, AsO_2_^−^, AsO_4_^3−^, Co^2+^, CrO_4_^2−^, Cu^2+^, Hg^2+^, Ni^2+^, Pb^2+^, TeO_3_^2−^, and Zn^2+^. Three mechanisms of resistance can be distinguished.

##### Proteins Conducting the Export of Heavy Metals

The first group of bacterial transport proteins is proteins associated with resistance, budding, and cell division (resistance-nodulation-cell division), shortly named “RND proteins”. These proteins were first found in the widely studied multi-resistant bacterium *Ralstonia metallidurans*, which are polyresistant to several heavy metals. This bacterium was isolated near a zinc smelter in Belgium and described by Mergeay et al. [44]. Besides the heavy metal efflux, RND proteins play a role in the budding of *Mesorhisobium lotti* and the cell division of *E. coli*.

RND proteins are divided into seven notable families found in organisms of all kingdoms. Most important are the families of inner-membrane, periplasmic, and outer-membrane efflux proteins. They form a single efflux—a protein complex that can evacuate Cd from the cytoplasm via the cytoplasmic membrane, cell wall, and outer membrane (typical for the Gram-negatives) into the extracellular space. Unlike the ATP-dependent one, this type of transport system is energy-independent, known as the CBA-efflux transport system.

**Figure 3:**
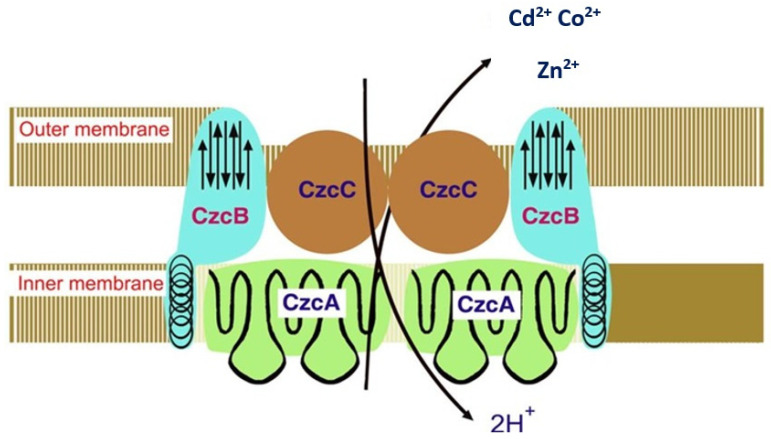
Czc model for Cd^2+^, Zn^2+^, and Co^2+^ efflux system functioning as proton/cation antiporter consisting of inner membrane (CzcA), outer membrane (CzcC), and membrane fusion (CzcB) proteins functioning as a dimer [45].

The Cd-Co-Zn- CBA-efflux system in polyresistant *R. metallidurans* is widely discussed. This beta-proteobacterium contains two large plasmids that determine its resistance to heavy metals, including Cd: pMOL30 and pMOL28. The first plasmid, pMOL30, encodes resistance to Co^2+^, Zn^2+^, and Cd^2+^ (named *czc* genes). When genes are expressed, they increase the MIC of Zn^2+^, Co^2+^, and Cd^2+^ from 7 to 50 times [37]. The structural genes responsible for resistance to Cd and other heavy metals in the pMOL30 plasmid were cloned and sequenced at least 20 years ago. Four open reading frames (ORFs) were detected in the czc-DNA sequence. They are arranged on the czc operon as follows: first is the *czcC* gene (encodes a CzcC protein of 346 amino acids (AA) followed by the *czcB* gene (CzcB protein composed of 521 AA), the *czcA* gene (CzcA protein composed of 1064 AA), and the *czcD* gene (CzcD protein consisting of 200 AA) [45]. The second plasmid, pMOL28, encodes resistance to Ni^2+^ and Co^2+^, increasing the MIC of Co^2+^ by 16 times. This type of resistance is called the *cnr* gene, which encodes the CnrA protein. CzcA and CnrA proteins were the first proteins identified in the RND family. The resistance to Cd, along with cobalt and zinc, in the bacterium *R. metallidurans* (former name *Alcaligenes eutrophus*) is shown in Figure 3.

##### Proteins Facilitating the Transport of Heavy Metals

These proteins (cation diffusion facilitators, or CDF) have been found in many prokaryotes and eukaryotes. Their substrate is predominantly cadmium but also zinc, cobalt, nickel, and iron cations [45]. Ion transport is carried out via both concentration and chemiosmotic gradients. Bacteria usually possess one or more CDF genes. Such have been found in the bacterium *R. metallidurans* and yeast *Saccharomyces cerevisiae*.

##### P-Type ATP-Ase

P-type ATP-ases play a protective role on the bacterial cell against heavy metals. These enzymes belong to the transport proteins that pump out from the cell the metal cations using energy from ATP hydrolysis, i.e., their action is energy dependent. Among the heavy metals, their substrates are the Cd and silver cations. P-type ATPase can import its substrate from the external environment into the cytoplasm and export it from the cytoplasm outside the cell [45]. According to this principle, P-type ATP-ases are divided into importing and exporting categories. It should be noted that P-type ATPase is precious for two reasons: it imports micronutrients such as Mg^2+^, but at the same time, it can also import heavy metals, and secondly, it exports heavy metal cations and thus neutralizes them, driving the bacterial cell to life.

#### 3.1.2. Bacteria in Cd-Contaminated Soil

In a study by Salam et al., Illumina shotgun sequencing of DNA extracted from two Cd-contaminated agricultural soils showed the predominance of the phyla, classes, genera, and species of *Proteobacteria* (37.38%), *Actinobacteria* (35.02%), *Prevotella* (6.93%), and *Conexibacter woesei* (8.93%) in the first sample, and *Proteobacteria* (50.50%), *Alphaproteobacteria* (22.28%), *Methylobacterium* (9.14%), and *Methylobacterium radiotolerans* (12.80%) in the second one. The study concluded that contamination with Cd has a significant impact on the structure and function of the soil microbial community, changes the resistome of heavy metals, modifies the physicochemical properties of the soil, and leads to the substantial decline of certain native community members that are not accustomed to Cd stress [46].

The presence of Cd has a crucial impact on bacterial diversity, and there are notable differences in microbial communities between areas with high Cd pollution and those with low Cd pollution. Yu et al. reported that higher concentrations of Cd significantly increased the abundance of *Proteobacteria* and *Gemmatimonas* and decreased the abundance of *Nitrospirae*. Members of the genera *Burkholderia* and *Bacillus* were reported to develop a resistance to Cd and may play an essential role in the bioremediation of Cd-contaminated soils [47]. In a study by Luo et al., high Cd-level sites displayed lower diversity indices than low Cd-level sites. The dominant phyla observed by the authors in paddy soil samples are *Proteobacteria*, *Chloroflexi*, *Acidobacteria*, *Actinobacteria*, *Gemmatimonadetes*, *Verrucomicrobia*, *Thaumarchaeota*, *Firmicutes*, and *Nitrospirae*. In this study, it is pointed out that *Actinobacteria* are tolerant to Cd, whereas *Proteobacteria*, *Verrucomicrobia*, and *Nitrospirae* are sensitive [48].

Sun et al. [49] found that the biodiversity and composition of the soil microbial community were affected in Cd-contaminated soil, with bacterial diversity impacted more than fungal diversity. The abundance of the soil microbial community decreased. At the same time, the composition changed at the phylum level, specifically in biomarkers for bacteria such as *Saccharibacteria* and *Gemmatimonadetes*, as well as in *Arenimonas*, *Xanthomonadales*, *Nitrosomonadaceae*, *Methylophilales*, *Caulobacteriales*, *Aeromicrobium*, *Chitinophagaceae*, *Acidimicrobiales*, *Nocardioidaceae*, *Propionibacteriales*, *Frankiales*, and *Gemmatimonadaceae*, which were found to be positively correlated with the total and available Cd [49]. Khan et al. describe an overview of Cd toxicity against living organisms and microbial resistance mechanisms, emphasizing the efflux systems, antioxidant profiling, and Cd eradication potential exhibited by microorganisms when exposed to Cd^2+^. Cd resistance and bioremediation potential make these microorganisms a good bioresource for green chemistry to exterminate environmental Cd^2+^ [50].

Heavy metals alter the soil microbial community composition, and the microorganisms that adapt to this stress increase in abundance [51]. Usually, the highest bacterial diversity is detected in severely contaminated soils [52]. As reported by authors, phyla *Proteobacteria* and *Acidobacteria* are abundant in soils contaminated by Cd and other heavy metals. The prevalence of *Actinobacteria*, *Acidobacteria*, *Proteobacteria*, and *Chloroflexi* in heavy metal-polluted soils was reported by Hemmat-Jou et al. [53]. Besides, the soil pH influences the heavy metals’ mobility and their toxic effects on bacterial communities. Acidic pH turns metals more bioavailable than neutral pH.

Using minimal bacteriological media with heavy metals added and no carbon source, researchers are able to grow, isolate, and further study the heavy metal-tolerant bacteria. To determine the bacterial sensitivity to heavy metals, growth of the bacteria is then assessed based on different criteria such as turbidity, biomass, and enzyme activities [54].

Several studies have shown that some lactic acid bacteria (LAB), including Lactobacillus plantarum, *Lactobacillus rhamnosus*, *Bifidobacterium breve*, and *Bifidobacterium lactis*, can bind and remove heavy metals such as Cd and Pb in vitro [55]. There is also substantial evidence that probiotic LAB have antioxidative properties that may be effective against Cd-induced oxidative stress in humans. Based on these unique characteristics, daily LAB consumption may be a preventive dietary strategy for people exposed to Cd [55]. Table 2 shows Cd removal potential by some bacterial and yeast strains.

### 3.2. Cd Accumulation and Toxicity on Fungi

The primary impact of Cd’s toxicity is mainly on fungi’s growth and replication. Fungi are particularly affected, as some fungal species may be eradicated following Cd exposure in soil. There is a selection pressure for resistant strains after low-level exposure to Cd in soil.

Soil fungi have an essential role in detoxifying and improving Cd-contaminated soils. The fungal cell wall contains polysaccharides and chitin and effectively helps control Cd tolerance and its uptake while acting as a barrier against Cd^2+^ entry into plant cells. Functional groups like carboxylic and hydroxylic groups and amino acids in the fungal cell wall carry a negative charge, enabling the cell wall to attract positively charged metallic ions. Research has shown that species such as *Absidia cylindrospora*, *Suillus luteus* (ectomycorrhizal fungi), and the group of *Neotyphodium endophytes* have significant potential for Cd tolerance and can be used effectively in remediating Cd-contaminated soils [56].

Heavy metals like Cd are known to be some of the most hazardous pollutants. In research conducted by Zheng et al., a filamentous fungus strain YZ1 was found in soil from wheat farmland. This strain, which belongs to *Purpureocillium* sp. based on its appearance and genetic evidence, had a minimum inhibitory concentration of 1 mM Cd and could survive in 100 mM Cd, reaching maximum biomass at 0.4 mM Cd. The strain YZ1 can be a great choice for cleaning up Cd-contaminated farmland in wheat-producing areas [57].

In another study by Fazli et al., seven fungi that were highly tolerant to Cd were tested: *Aspergillus versicolor*, *Aspergillus fumigatus*, *Paecilomyces* sp.9, *Paecilomyces* sp.G, *Terichoderma* sp., *Microsporum* sp., and *Cladosporium* sp. [58]. The findings revealed that the fungi displayed various resistance mechanisms against Cd and could sequester Cd from liquid environments. *Aspergillus versicolor* showed a remarkable difference in detoxification behavior compared to the other isolated fungi. It demonstrated a strong ability to grow actively in the presence of Cd and reduce the Cd concentration to less toxic levels. The introduction of *Aspergillus versicolor* as a scavenger organism marks the initial step in the emergence of this fungus in bioremediation science [58].

### 3.3. Cd Accumulation and Toxicity on Edible Mushrooms

The accumulation of heavy metals in the fruiting bodies of mushrooms is extensively studied. The mean content of Cd in analyzed mushrooms ranges from 0.370 to 2.151 mg/kg d.w., while Pb is found at the level of 0.243–0.424 mg/kg d.w. Heavy metals are believed to cause pronounced toxicological harm to human health when contaminated mushrooms, even at low metal concentrations, are consumed [59]. Furthermore, consuming mushrooms contaminated with heavy metals can lead to damage to the kidneys and heart, as well as the impairment of the digestive, immunological, skeletal, and nervous systems [59]. Mushrooms can gather high amounts of minerals, even when grown in soils with low metal content. This is attributed to the species’ genetic characteristics, including many transport genes and binding ligands [60].

Mushrooms can absorb specific forms of heavy metals, such as Cd^2+^, Cd^6+^, Hg^2+^, As^5+^, etc., into their fruiting bodies. In connection, metals’ intracellular speciation and uptake are typically regulated by metallothioneins and GT complexes that are directly linked to fungal physiology [61]. Species with the ability to accumulate Cd include *Agaricus bisporus*, *A. campestris*, *A. macrosporus*, *Armillaria mellea*, *Amanita muscaria* and *A. allies*, *Boletus edulis*, *Cantharellus cibarius*, *Cystoderma carcharias*, *Macrolepiota procera*, *Xerocomus badius*, and *Tricholoma matsutake*. The concentration of Cd in edible mushrooms can go up to 1.3925 mg/kg and readily accumulate in more significant quantities within the caps.

The highest allowable levels of Cd for *Agaricus bisporus* (common mushroom), *Pleurotus ostreatus* (oyster mushroom), and *Lentinula edodes* (shiitake mushroom) are set at 0.20 mg/kg. For other mushroom species, it is 1.0 mg/kg. The mentioned maximum levels are applicable after washing the mushrooms and separating the edible part [62].

As the number of wild mushroom consumers continues to rise, monitoring the presence of toxic elements and their potential risks has become important. In this regard, the elevated Cd levels in wild edible mushrooms can pose health risks to consumers, especially as several species of *Tricholoma* mushrooms are consumed in fresh or processed form [61].

### 3.4. Cd Accumulation and Toxicity on Plants

In plants, Cd predominantly accumulates in the roots, with lesser amounts in the leaves [63]. Cd exhibits toxicity to a broad range of plants, but its harmful effects are mitigated by sediment, high concentrations of dissolved salts, or organic matter. Cd adversely affects plant growth in experimental settings, although no field effects have been reported. Plants more readily take the metal from nutrient solutions than from soil, with most studies demonstrating its effects in nutrient solution cultures. Cd has been shown to impact stomatal opening, transpiration, and photosynthesis in plants grown in nutrient solutions [63].

Chlorosis and stunted growth in plants are easily identifiable indicators of Cd poisoning. Higher toxicity slows plant growth and causes necrosis [64]. Cd toxicity harms plants by limiting carbon fixation and reducing chlorophyll concentration and photosynthetic activity [65]. Cd accumulation in plants can result in various physiological, biochemical, and structural alterations. Its accumulation modifies mineral nutrient intake, slows stomatal opening by interfering with plant water balance, disrupts Calvin cycle enzymes, photosynthesis, and carbohydrate metabolism, changes antioxidant metabolism, and reduces agricultural output [66].

Exposure to Cd in the soil causes osmotic stress in plants by reducing leaf-relative water content, stomatal conductance, and transpiration, resulting in physiological damage [67]. Cd poisoning generates an overproduction of reactive oxygen species (ROS), which damages plant membranes and destroys cell proteins and organelles [65].

As reported by Huybrechts et al., exposing *Trigonella foenum-graecum* seeds to Cr, Pb, and Cd solutions revealed that Cd at 10 mg L^−1^ showed the most significant inhibitory effect on germination, the highest concentration tested. Similarly, *Triticum aestivum* required less Cd than Pb to hinder seed germination. However, it is important to note that there can be significant variations between plant species [64].

Cd is recognized for inhibiting seed germination through various mechanisms [68]. In *Vigna unguiculata* seeds, the inhibitory effect of Cd was suggested to be caused by impaired water uptake, limiting the water availability for the developing embryo. In addition to a restricted water supply, inhibition of starch mobilization from the endosperm, combined with an impaired translocation of soluble sugars to the embryonic axis, can further starve the embryonic axis. A decrease in hydrolyzing enzymes, including α-amylase, proteases, and acid phosphatases, in *Sorghum bicolor* seeds was proposed to be responsible for the reduced storage mobilization. Calcium is crucial for amylase activity, and replacing the chemically similar Cd ion could disrupt normal enzyme functioning. Furthermore, radish seeds experienced direct competition for Ca-calmodulin binding sites between Ca and Cd ions [64].

Cd exposure is widely known to cause DNA damage. The mechanisms behind this Cd-induced DNA damage involve the ROS-induced formation of 8-hydroxyguanosine and the inhibition of DNA repair systems. Table 3 describes Cd’s effect on different plant species.

The adverse effect of Cd on the cell cycle is presented in Table 4.

The structure that provides the primary defense for a plant cell against pathogen attacks and adverse environmental conditions such as drought and metals is the cell wall. Preventing excess Cd from entering the cytoplasm is crucial because it can cause damage to macromolecules, proteins, and DNA due to Cd-induced oxidative stress. Roots in direct contact with Cd from the soil have cell walls that play a crucial role in this process. Research indicates that most plant species store the majority of Cd within the cell walls of roots [64]. When the capacity of the cell wall is surpassed, Cd may form complexes with PCs and then can finally be sequestered *within vacuoles* by transferring ABC transporters through the tonoplast.

For a plant to go through its life cycle, it needs to start the reproductive phase, which in higher plants involves the development of flowers, pollination, and fertilization, followed by seed production. When *Brassica campestris* plants were exposed to Cd during the flowering stage, the content of glutathione (GSH) and ascorbate (AsA) decreased the most, suggesting that the reproductive phase was highly vulnerable [64]. Cd has been demonstrated to harm pollen germination and disrupt pollen tube morphology in various plant species [69].

### 3.5. Cd Accumulation and Toxicity on Animals

Cd toxicity causes a wide range of health issues, including some of the deadliest diseases, such as heart disease [70,71], kidney disease [72,73], liver disease [72], cancer, and diabetes [70]. Practically every system in an animal’s body can be harmed by Cd. Over an extended period, Cd accumulates in the kidney and liver [74]. Farm animals can come into contact with contaminated water, soil, vegetation, and car and industrial emissions [75,76]. Foods of all kinds contain a lot of different types of Cd. Foods made from grains, leafy vegetables like spinach, and mainstays like potatoes have relatively high levels of Cd, ranging from 30 to 150 ppb [77]. Sunflower, soybean, and peanut seeds all have naturally high levels of Cd, seemingly with no adverse health effects. Fish and meat typically have lower levels of Cd, ranging from 5 to 40 ppb [78]. Offal organs where Cd accumulates, like the kidney and liver, can contain remarkably high Cd levels—up to 1000 ppb [78].

Numerous organisms readily accumulate Cd, notably mollusks, where bioconcentration factors can reach several thousand. Soil invertebrates also exhibit significant Cd accumulation. In contrast, most organisms display low to moderate concentration factors, typically under 100 [79]. Cd in tissues is often bound to proteins, including specific heavy-metal-binding proteins known as metallothioneins, which have been isolated from organisms exposed to Cd [80]. The highest Cd concentrations are found in the kidney, gills, and liver (or their equivalents). The primary route of Cd elimination in organisms is likely through the kidney, although in crustaceans, substantial amounts can also be shed via the exoskeleton.

The acute toxicity of Cd to aquatic organisms varies considerably, even among closely related species, and is influenced by the free ionic concentration of the metal. Cd interferes with calcium metabolism in animals, causing hypocalcemia in fish by inhibiting calcium uptake from the water. However, high calcium concentrations in the water can protect fish from Cd uptake by competing at the uptake sites [81]. Zinc exacerbates Cd toxicity in aquatic invertebrates. Sublethal effects on aquatic invertebrates include impaired growth and reproduction and structural damage to gills. Fish, particularly salmonids, show variable sensitivity to Cd, with sublethal effects, including spinal malformations [82]. Embryos and early larvae are the most susceptible life stages, while eggs are the least affected. There is no consistent interaction between cadmium and zinc in fish.

In a recent study by Djedjibegovic et al., Cd content (mean concentration) was tested in seafood samples in Bosnia and Herzegovina. The team concluded that mercury and cadmium were detected in all analyzed samples (100%), while lead was detected in 33 samples (89.2%). Metals content was in the order Hg > Cd > Pb in most of the species, except blue mussel (Pb > Cd > Hg) and Indian white prawn (Hg > Pb > Cd). Cadmium content was close to the corresponding MRL in two samples of Patagonian squid (0.918 and 0.896 mg kg^−1^). It was also relatively high in the other three samples of the same species (0.591, 0.425, and 0.391 mg kg^−1^) [83].

Exposure to Cd at relatively low doses impairs sperm motility and morphology and can reduce male fertility in rats [84]. In a study conducted in the Czech Republic by Drapal et al., higher average levels of Cd were found in the liver (0.10 mg/kg) and kidney (0.62 mg/kg) of cattle over 2 years old compared to lower levels in the liver (0.06 mg/kg) and kidney (0.24 mg/kg) of cattle under 2 years old. Ruminants are exposed to Cd contamination by consuming pasture during the summer and preserved feed, including cereals, during the winter [85,86]. According to Chirinos-Peinado et al., Cd and Pb can accumulate in fresh cow’s milk [87]. Fay et al. discussed that Cd nephrotoxicity is associated with altered microRNA expression in the rat renal cortex [88].

### 3.6. Cd Accumulation and Toxicity in Humans

Cd poses a significant health danger to people, even at low concentrations. The body’s ability to adapt to Cd exposure is limited due to its inability to undergo metabolic degradation and poor excretion [14]. Cd toxicity affects various animal organs, including the liver, kidney, lungs, testes, prostate, heart, skeletal system, neurological system, and immune system. Prolonged exposure to Cd can accumulate in the body and cause illnesses primarily affecting the lungs and kidneys [89]. It is believed that chronic lung diseases, high blood pressure, cancer, leukemia, genetic toxicity, damage to human organs, abdominal pain, burning sensations, nausea, vomiting, and various cancers may be related to slow poisoning by Cd in small doses. Additionally, Cd exposure may be associated with diseases related to the central nervous system, cognitive and behavioral functions, chronic diseases, teratogenic effects, cardiovascular disease, lung function abnormalities, and damage to the kidneys [90].

Acute Cd poisoning symptoms typically develop after 24 h and include shortness of breath, weakness, and fever. Cd poisoning can lead to pulmonary edema, pneumonia, and, in severe cases, respiratory failure, and death. Cadmium’s distribution in the body is determined by its chemical form. Exposure to Cd in the form of inorganic salts (e.g., CdCl_2_) leads to a higher accumulation of Cd^2+^ ions in the liver, kidneys, and bones than Cd combined with metallothionein. CdCl_2_ primarily accumulates in the liver, and CdMT accumulates in the kidney [91]. Cd is deposited in various organs, including the liver, kidney, testis, spleen, heart, lungs, thymus, salivary glands, epididymis, and prostate. However, because of their high MT content, the liver and kidney store around 50% of the accumulated Cd [92]. Cd can accumulate in the pancreas, lungs, central nervous system, and testes in men. Cd particles are carried through the primary olfactory neurons [93].

#### 3.6.1. Cd Distribution in the Human Body

**Figure 4:**
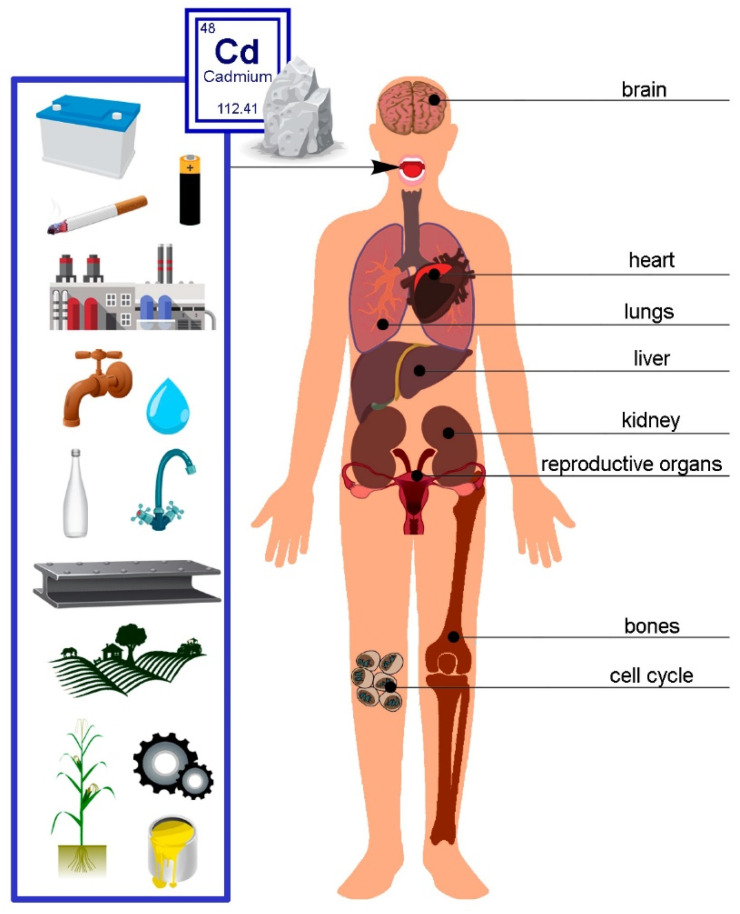
Sources of Cd and its most significant effects on different parts of the human body [90].

Cd, a subject of increasing scientific and medical interest due to its detrimental effects on health, presents a pressing need for a comprehensive understanding of its distribution in human tissues and organs. This knowledge forms the basis for crucial in vitro and in vivo animal studies [94]. The specific amounts of Cd in various cell types and tissue layers, in particular, have not been thoroughly explored. This underscores the necessity for further investigation [95]. Only a handful of researchers have dealt with the total Cd content in the human body [94]. Figure 4 below presents the primary sources of Cd and its effects on the human body.

##### Ingestion

Cd tends to persist and accumulate in soil, eventually entering plant metabolism. Cd enters the food chain as it accumulates in edible plant parts such as fruits and seeds [96]. This accumulation rises when soil pH decreases; therefore, acid rain has the effect of raising Cd concentrations in plants. Cereals and bread account for 34% of daily Cd intake in Western countries, followed by leafy vegetables, particularly spinach, among adults (20%), potatoes (11%), legumes and nuts (7%), stem/root vegetables (6%), and fruits (5%) [97]. Mei et al. studied the accumulation and molecular mechanisms of Cd in cereal crops and tobacco [98]. Wheat can also accumulate high levels of Cd in the grain [99]. In Eastern countries, fish and shellfish are the most common Cd sources, followed by grains and vegetables, particularly rice [100]. Rice is one of the most important food crops worldwide, feeding over 5 billion people. Thus, Cd pollution in rice has attracted great attention [101]. Although dietary Cd exposure poses a minor health risk in most Eastern countries, it remains a concern for specific subgroups. Cd is dispersed over the earth, and there are locations with extremely high concentrations of Cd in the soil. Crop uptake of Cd in these locations can result in high food exposures for those living nearby. For example, in Japan’s Jinzu and Kakehashi river basins, soil is extensively contaminated with Cd from industrial waste [102,103]. Locals who habitually ingested rice cooked with Cd-contaminated water experienced a severe kidney and bone illness known as “itai-itai” sickness, which was marked by bone distortion and many fractures, particularly in women [104]. The decrease in Cd intake over the last 50 years could be ascribed to less sewage sludge escaping into agricultural soil due to improved control and environmental awareness in industrialized countries. These processes primarily transferred Cd from plants into the food chain, increasing human exposure to the metal. Some aquatic creatures can also be significantly impacted by Cd deposition at levels above the regulatory limits [82].

Cd intake from meals in humans ranges from 8 to 25 µg per day, with only 0.5 to 1.0 µg retained in the body. Factors affecting this form of absorption include dose, exposure period, dietary components, nutritional status, age, and gender [93]. As reported by Raikwar et al., the safe recommended intake of Cd is 15–50 µg/day for adults and 2–25 µg/day for children [74].

##### Inhalation

**Figure 5:**
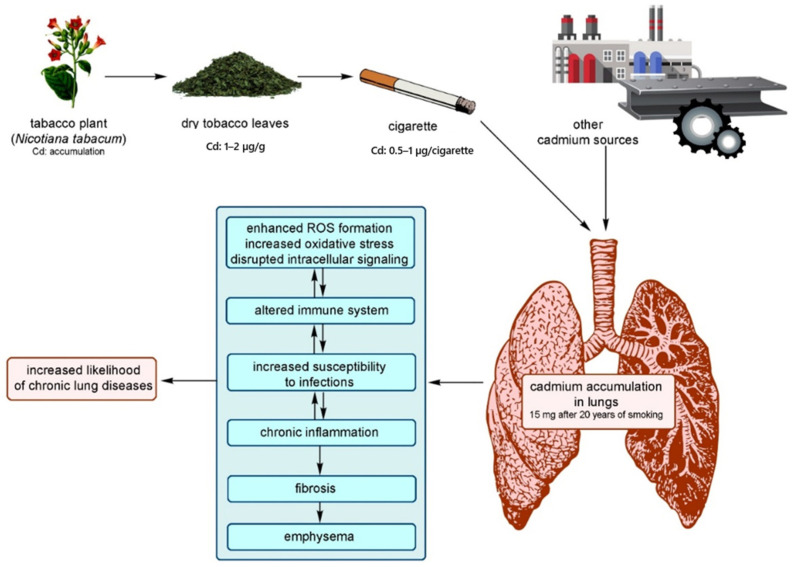
Cd exposure leads to the development of smoking-related lung diseases [90].

Cd air levels can be hundreds of times higher in the workplace than in the broader environment [105]. The Occupational Safety and Health Administration (OSHA) sets the permissible exposure limit (PEL) for Cd fumes or Cd oxide in the workplace at 0.1 mg/m^3^. However, Cd concentrations in ambient air range from 1 × 10^−6^ mg/m^3^ in non-industrialized areas to 4 × 10^−5^ mg/m^3^ in urban areas. Non-occupational Cd exposure from the air is unlikely to cause harmful health effects. Cd air levels are typically insufficient to create health issues in the general population. Even in places with substantial industrial Cd emissions, the average atmospheric concentration does not exceed 35 ng Cd/m^3^ of air [106]. Tobacco smokers have four to five times greater levels of Cd in their blood and two to three times higher amounts in their kidneys than nonsmokers (Figure 5) [107,108]. According to Gray et al., the liquids used in electronic cigarettes also contain Cd with a concentration of 0.108 µg/g [109].

##### Permeation

**Figure 6:**
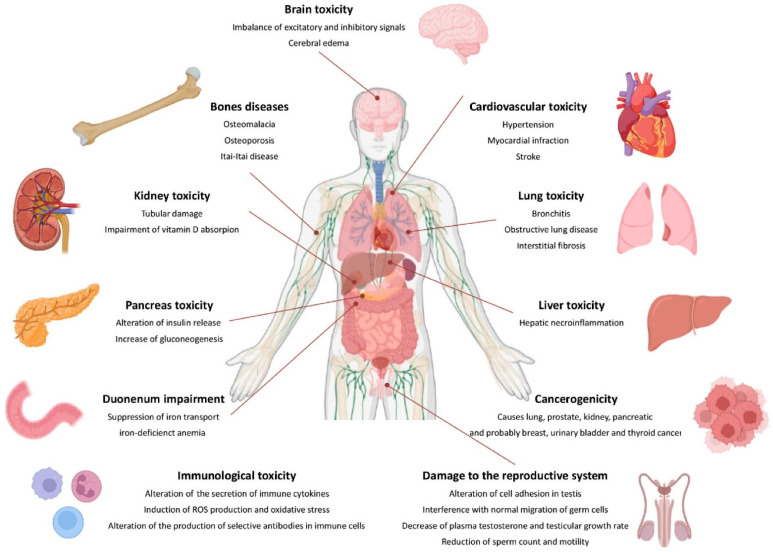
Primary outcomes in health effects following chronic Cd exposure [1].

The skin absorbs tiny amounts of Cd. Hence, it is not considered a critical route of exposure. However, recent research has highlighted the environmental significance of photosensitive CdS and CdSe pigments and nano semiconductors, whose oxidized products (cadmium sulfate (CdSO_4_) and cadmium selenite (CdSeO_4_)) are significantly more soluble and bioavailable, and thus potentially more dangerous [110]. An in vitro investigation employing human full-thickness skin as a model to describe the impact of Cd exposure on skin revealed that the metal only enters the epidermis; previously, it was demonstrated that its solubility into the *stratum corneum* layer is a rate-limiting process (Figure 6) [92].

#### 3.6.2. Harmful Effects on Human Organs and Systems

Cd can act through molecular mimicry, which occurs when metals attach to nucleophilic sites on specific biological molecules, forming complexes that can structurally and/or functionally resemble endogenous substrates that typically bind to the active sites of carrier proteins, channels, structural proteins, enzymes, and/or transcription factors. In principle, ionic mimicry shares similarities with molecular mimicry. Generally, the term ionic mimicry refers to the capacity of an unbound, cationic species of metal to act as a structural and/or functional counterpart or mimic of another (usually an essential) element at the site of a carrier protein, ion channel, enzyme, structural protein, transcription factor, and/or metal-binding protein. For instance, considerable evidence has been gathered demonstrating that the cationic forms of certain toxic metals, such as Cd, can utilize ion channels (especially Ca^2+^ channels) and specific membrane transporters, like the divalent metal transporter 1 (DMT1/DCT1/Nramp1), to enter target cells in mammalian organisms [111]. Cd^2+^ can interact with membrane transporters responsible for the uptake of essential metals like Ca^2+^, Fe, and Zn, allowing it to enter target cells in organs negatively impacted by this metal. This process occurs through ionic mimicry, where Cd^2+^ imitates the divalent cationic forms of these essential metals at the binding sites of various carrier proteins and/or channels that facilitate their transport [112].

##### Effects on the Blood and Circulatory System

Smokers have higher levels of Cd in their blood and are more likely to develop atherosclerosis, specifically peripheral vascular disease, than non-smokers [113]. Research suggests that low-dose Cd exposure increases the incidence of peripheral arterial disease (PAD). This element also has a detrimental effect on the cardiovascular system [114]. Cd’s harmful effects on the vasculature of many organs, particularly endothelial cells, have been demonstrated through experimental research. Cd damages endothelium and smooth muscle cells, leading to the production of atherosclerotic plaques [115]. This is supported by epidemiological and clinical research. Cd can also influence homocysteine metabolism. High levels of homocysteine can lead to heart disease, stroke, peripheral vascular disease, and cognitive impairment [116].

##### Effects on the Reproductive System

Cd can impair reproductive functioning. Exposure to Cd poisoning predominantly impacts testicular function [117]. The toxic effects of Cd in the testis include damage to the vascular endothelium. Cells experience morphological and functional changes, including inhibition of testosterone synthesis and impaired spermatogenesis due to oxidative stress, impaired antioxidant defense mechanisms, and inflammatory response severity. Cd can disrupt prostate function, affecting hormonal activity, secretion, and fertility in men [93]. Exposure to Cd harms human male reproductive organs/systems, impairing spermatogenesis, semen quality, particularly sperm motility, and hormone synthesis/release [118]. According to experimental and human investigations, it also disrupts female fertility, reproductive hormone balance, and menstrual cycles [119]. Based on the available data, it is possible to conclude that low-dose Cd exposure harms both male and female reproduction and impacts pregnancy or its outcome [120]. Furthermore, maternal prenatal Cd exposure may have a distinct effect on male and female offspring, particularly female offspring. As a result, measures must be taken to limit exposure to Cd [121]. The testicles are the primary target of Cd-induced acute toxicity [122]. The effect is quick. The testicles begin to shrink, followed by inflammation, edema, acute bleeding, and necrosis in less than 24 h after a single dose of cadmium [123].

Even a signal injection of Cd^2+^ can cause acute testicular necrosis, as reported by Bridges and Zalups [111]. In testes, Cd^2+^ mimics Zn^2+^ using a Zn^2+^ transporter, which contains many amino acids. In the same paper, it is shown that Cd^2+^ uptake occurs via passive and active mechanisms of interstitial cells of rat testes. Fe^2+^ is also involved in the Cd^2+^ uptake in the testes via the Fe^2+^ transporter DMT1, which is identified in the Sertoli cells of the testes.

Cd^2+^ can also mimic estrogen (estradiol). Twenty-four-hour exposure to CdCl_2_ leads to the same physiological effect when treated with estradiol, both activating the estrogen receptor proven in a breast cancer cell line (MFC-7). As Cd^2+^ does not exist in an unbound state in physiological solutions, data obtained from studies in which cells were treated with CdCl_2_ may not accurately represent physiological events in vivo [111].

##### Effects on the Respiratory System

Inhalation produces respiratory distress and damages the respiratory tract [124]. High Cd concentrations in contaminated air have been linked to conditions like emphysema, anosmia, and chronic rhinitis. Lampe et al. [125] studied the impact of Cd exposure on lung function among 96 males with one to three lung function tests between 1994 and 2002. Researchers discovered that smoking was linked to lower forced expiratory volume in 1 s (a measure of lung function) and higher urine cadmium levels [125]. Inhaling Cd can cause respiratory distress syndrome [93].

##### Effects on the Kidney System and Bones

Long-term exposure to high doses of Cd results in itai-itai disease, primarily affecting women. This disease is marked by severely compromised tubular and glomerular function, generalized osteomalacia, and osteoporosis, leading to multiple bone fractures [126]. Prolonged exposure to low doses of Cd has been associated with tubular damage, resulting in reduced reabsorptive capacity for nutrients, vitamins, and minerals; nephropathy; and proteinuria [77,107]. Non-absorbed molecules include zinc or copper bound to metallothionein (MT), glucose, amino acids, phosphate, calcium, and low-molecular-weight (LMW) proteins like 2-microglobulin (2-M) and 1-microglobulin (1-M), also known as protein HC, retinol-binding protein (RBP), and uric acid, resembling Fanconi syndrome, a genetic disorder of renal tubular transport. Urinary markers for Cd exposure include Cd itself, LMW proteins (2-M, 1-M), and enzymes of renal tubular origin, such as *N*-acetyl glucosaminidase (NAG). Generally, urinary Cd levels indicate long-term body burden before kidney damage develops, while blood Cd levels indicate recent exposure [127]. Cd disrupts the metabolism of calcium, magnesium, iron, zinc, and copper in cells, leading to demineralization, osteomalacia, osteoporosis, and bone disorders, necessitating the replacement of these ions. Cd’s competitive displacement of calcium ions weakens bone structure, often causing fractures, especially in children and postmenopausal women. Cd also inhibits the activity of 1-hydroxycholecalciferol hydroxylase, an enzyme essential for converting 25(OH)D3 to the active form of vitamin D3, 1,25(OH)_2_D3, in the kidney [91]. This active form is crucial for calcium absorption in the intestine [93]. Research of 31- to 60-year-old men from Pakistan’s growing industrial nation found that diabetic males (N = 196) had considerably higher Cd levels in their blood and urine than non-diabetic males (N = 238). These disparities were observed in both smokers (N = 209) and non-smokers (N = 225) [128]. Although Cd is known for its renal toxicity, the dose-response relationship at low environmental exposure levels remains unclear. The Centers for Disease Prevention and Control (CDC) and European health agencies acknowledge this uncertainty and the potential public health impact of low-level Cd exposure, urging further research.

The Avonmouth lead/zinc smelter in southwest England closed in early 2003 and was the UK’s largest source of atmospheric Cd emissions. It emitted 978 kg of Cd in its final year, accounting for nearly 30% of the UK’s point source emissions. The site had produced zinc for over 70 years, emitting other nephrotoxic metals like lead, mercury, and arsenic. Given Cd’s high nephrotoxicity and long biological half-life, this study focused on Cd exposure. With approximately 50,000 people living within 5 km of the smelter, there is concern about increased Cd exposure from inhalation contaminated air and ingesting homegrown vegetables and house dust. A total of 180 volunteers (74 men and 106 women) were studied for health problems caused by Cd. Among the participants, 109 (40 men and 69 women; 61%) were never-smokers, 36 (19 men and 17 women; 20%) were former smokers, and 32 (13 men and 19 women; 18%) were current smokers. Results obtained by urine analyses showed early kidney damage [129].

The kidney is one of the main organs that suffer negatively in humans after long-term oral or inhalation exposure to Cd^2+^. The harmful impacts of Cd^2+^ on the kidneys became evident when factory workers involved in the production of nickel-cadmium batteries were exposed to cadmium oxide dust and cadmium vapors. The kidney function of these workers experienced notable changes, leading to proteinuria and a decreased glomerular filtration rate [111].

The majority of Cd^2+^ found in the kidney is concentrated in the epithelial cells of the proximal tubule. Due to Cd^2+^’s strong binding affinity for thiol-containing biomolecules like GSH and Cys, the forms of Cd^2+^ likely presented to the luminal membrane of proximal tubular cells are complexes of these molecules, such as G-S-Cd-S-G or Cys-S-Cd-S-Cys. Furthermore, given that γ-glutamyltransferase and cysteinylglycinase are highly abundant in the luminal plasma membrane of proximal tubular cells, it seems unlikely that G-S-Cd-S-G is absorbed as an intact complex [111]. In vivo research in rats has shown that after administering CdCl_2_ subcutaneously with excess Cys, increased amounts of Cd accumulate in the epithelial cells of the proximal tubule. At least one mechanism for renal uptake of Cd^2+^ exists at the luminal side and another at the basolateral side. In addition to other pathways, Cd^2+^ might gain entry into proximal tubular cells via Ca^2+^ channels, though this theory lacks definitive evidence. Nonetheless, studies have indicated that Cd^2+^ can use Ca^2+^ channels in isolated cells from different organs, such as the liver and intestine [111].

##### Effects of the Liver and Intestines

Following oral intake, Cd^2+^ is absorbed by the intestines and transported to the liver through portal blood, where hepatocytes readily uptake it. After administering Cd^2+^ intravenously, up to 60% of a non-toxic dose (5 μmol/kg) can be found in rat livers within an hour. In studies using low doses of CdCl_2_, G-S-Cd-S-G, or Cys-S-Cd-S-Cys, less than 1% remained in the bloodstream after one hour, demonstrating the swift extraction by the liver and other tissues [111]. Approximately 50% of the Cd^2+^ still present in the blood is associated with cellular components, mainly within erythrocytes, likely via an anion exchanger. One membrane transporter that is probably responsible for the sinusoidal uptake of the cationic version of Cd^2+^ is DMT1. This transporter has been isolated and identified as a proton-coupled iron transporter. In liver cells, DMT1 is situated in the sinusoidal membrane [111].

The uptake of Cd^2+^ by hepatocytes may involve calcium channels. Given that the ionic radius of Cd^2+^ closely resembles that of Ca^2+^, Cd^2+^ can likely imitate Ca^2+^ at calcium channels to enter hepatocytes. In fact, in vitro research using primary cultures of rat hepatocytes and cultured immortalized hepatocytes shows that Cd^2+^ may be transported via calcium channels. These studies found that the uptake of Cd^2+^ into hepatocytes was significantly blocked by Ca^2+^ channel antagonists, such as diltiazem and verapamil. Cd^2+^ entered these cells through voltage-gated L-type calcium channels. Moreover, additional experiments where hepatocytes were treated with Ca^2+^ channel antagonists indicated that around one-third of the Cd^2+^ that entered the cells did so through calcium channels [111].

The duodenum and the initial portion of the jejunum account for most of the absorption of ingested Cd^2+^. The duodenum is a key area for the absorption of Fe^2+^. The similarity in ionic radii between Cd^2+^ (0.95 Å) and Fe^2+^ (0.55 Å) implies that these two cations might share some transport mechanisms. In vivo studies in rats reveal that Cd^2+^ disrupts the intestinal uptake of Fe^2+^, indicating that these two metal ions could use similar transport pathways. DMT1, found in the luminal plasma membrane of enterocytes, seems to play a crucial role in the intestinal absorption of Fe^2+^. In another research, rats were fed a diet deficient in Fe or a diet supplemented with Fe, following which they were given an oral dose of CdCl_2_. The mRNA levels of DMT1 in the small intestine of the rats fed the Fe-deficient diet were 15-fold greater than those in the ones fed the supplemented diet [111]. The content of Cd^2+^ in the small intestines of animals with depleted Fe stores was significantly greater than that in rats fed a supplemented diet. Thus, it can be concluded that the Fe status significantly affects the expression of DMT1. Ca^2+^ channels in the luminal plasma membrane can serve as a way for Cd^2+^ to access the cytosolic compartment of enterocytes. The paper by Bridges and Zalups describes it as a two-step mechanism of Cd^2+^ transport across the luminal plasma membrane of enterocytes. The first is the binding of Cd^2+^ to the plasma membrane. The binding was susceptible to EDTA but insensitive to temperature, whereas the second step was sensitive to temperature and insensitive to EDTA. The same paper highlighted that duodenum was rich in amino acids and peptides that can absorb significant amounts of Cd^2+^. In contrast, Cd^2+^ can evacuate from the basolateral membrane of the enterocytes via Zn^2+^ transporter (ZnT-1), which was demonstrated by in vivo and in vitro studies. Based on these statements, it can be concluded that Cd^2+^ is a competitive inhibitor of Ca, Fe, and Zn [111].

##### Effects on the Central Nervous System

Accumulating evidence over the years suggests that Cd may be a potential cause of neurodegenerative diseases. This could be related to the excessive production of free radicals, which damage organs depending on their defense mechanisms [130]. Since Cd is a toxic agent affecting various cell types, this study aimed to investigate Cd’s effects and consequences on different organs in mice. To test the hypothesis of concentration-dependent reactive oxygen species (ROS) generation and DNA damage, Agnihotri et al. [130] assessed the serum of 4–5-week-old male Swiss albino mice, which were treated with cadmium chloride (CdCl_2_), added to their drinking water for 30 days. The expression of Bcl-2-associated X protein (Bax), an apoptotic marker, was twice as high in the brain compared to the liver at an exposure level of 0.5 mg L^−1^ CdCl_2_ [131]. Additionally, correlation and linkage data analysis of the antioxidant defense system showed rapid changes in the brain compared to other organs in the study. The results indicate that even at low doses, Cd impairs the brain due to its lipid peroxidase sensitivity, which promotes Cd-induced oxidative injury in the brain [132]. Human studies indicate that exposure to Cd can lead to aberrant behavior and lower IQ in children and adults. The blood-brain barrier is susceptible to Cd. Cd has direct harmful effects when it crosses the blood-brain barrier, which can happen in young children or with certain medical conditions. The choroid plexus epithelium can accumulate significant levels of Cd, which reduces concentrations in other body regions [133].

Cd toxicity can cause cell injury, cell death, and organ failure through various chemical pathways, the most common of which is oxidative stress, in numerous body compartments and tissues, including the central nervous system [134]. In this context, many in vivo and in vitro research studies have provided evidence showing Cd-induced neurotoxicity in the CNS and Cd’s involvement in many compartments and cells of the CNS [135]. Cd-treated embryos developed a smaller head with unclear boundaries between the brain subdivisions, particularly in the mid-hindbrain region. Embryos display normal anterior-to-posterior regionalization; however, the commitment of neural progenitor cells was affected by Cd [81]. Cd has been shown to produce free radicals in the brain, potentially damaging neurons and oligodendrocytes (OLG). OLGs are the glial cells which myelinate axons in the CNS. An early study reported that Cd toxicity affected CNS white matter [136], and one laboratory demonstrated that OLGs are direct targets of this structure [137]. Experimental studies have shown that Cd can also be a potent neurotoxicant for the peripheral nervous system [138]. Moreover, Cd has a half-life of more than 15 years in humans. Elderly workers may be more susceptible to an increased Cd body burden and may develop peripheral polyneuropathy (PNP) over time [139]. There is significant evidence suggesting that Cd^2+^ can be absorbed by the distal segments of the nephron. Additionally, Ca^2+^ channels could offer a pathway for the uptake of luminal Cd^2+^ in the cells that line the distal nephron [111].

##### Effects on the Immune System

Chronic Cd exposure can negatively impact the immune system [140]. Cd targets several cells, including T cells, macrophages, B cells, and natural killer cells. Cd’s direct immunotoxicity alters immunological responses, including cell-mediated and humoral immunity. Cd toxicity may cause anemia and eosinophilia, according to Sarkar et al. [93]. The effect of Cd on the immune system has been investigated utilizing experimental animals, particularly rodents. Cd can interact directly with immune system cells, causing modifications in their status and functionality that can be evaluated in vitro. Direct consequences include cell death and signaling pathways interference, altered cytokine production, cell surface marker expression, cell activation, and differentiation [141]. It has been demonstrated that Cd exposure impairs immune cells’ ability to operate normally. When administered orally to mice, Cd increases vulnerability to herpes simplex virus type 2, reduces T and B cell production, and enhances macrophage phagocytosis [142]. It has been shown that school-age children exposed to Cd experience a drop in sensitivity and IgG antibody titers. Stress is thought to be the cause of the induction of apoptosis, as is the case with many other immunotoxic drugs. Cd also has an impact on cytokine production [143].

## 4. Molecular Mechanisms of Cd Toxicity

Cd affects cellular proliferation, differentiation, and apoptosis. The International Agency for Research on Cancer (IARC) classed it as a proven carcinogen, a member of Group No. 1. However, it has a low genotoxic potential. Cd’s indirect effects cause reactive oxygen species (ROS) production and DNA damage [144,145]. In vitro studies indicate that Cd has several activities that still need to be fully understood. Chronic heavy metal exposure leads to increased expression of stress proteins (such as heat shock protein 70 and metallothioneins), which can cause apoptosis, growth inhibition, proliferation, or carcinogenicity in animal cells, depending on factors like amount, timing, cell line, and presence of other chemicals. Cd carcinogenesis is primarily caused by oxidative stress, DNA repair inhibition, and altered apoptosis rates [93,146]. A recent study describes the effects of Cd on signaling through Ca^2+^, NO, c-AMP, nuclear factor kappa-light-chain-enhancer of activated B cells (NF-κB), and developmental pathways such as Wnt signaling, as well as kinases [132]. There are well-established effects of Cd on kinase activation and downstream events of immediate early response oncogene induction, as these events are likely involved in cancer promotion and progression and cell survival [147]. Mitochondria regulate cell homeostasis, proliferation, motility, senescence, and death. Cell and tissue aging, as well as numerous illnesses, such as Alzheimer’s disease, Parkinson’s disease, Huntington’s disease, and even cancer, are associated with mitochondrial dysfunction [148].

Cd can change the expression of various genes, including immediate early response genes, stress response genes, transcriptional factors, and translational factors. It activates the c-jun N-terminal kinase (JNK) pathway, leading to the over-expression of genes responsible for the synthesis of metallothionines and heat shock proteins. Cd also affects transcription factors such as metal-regulatory transcription factor, nuclear factor-κB, and NF-E2-related factor 2, as well as translational factors like TIF3 and TEF-1δ. These changes can provoke the development and progression of tumors [149].

The mechanisms by which Cd disrupts gene expression include changes in Ca intracellular level, ROS generation, effect on cell kinases, and DNA methylation. Cadmium increases intracellular Ca content, affecting gene expression directly by binding to target sites in different genes or indirectly through activation of kinases. Additionally, it mimics Ca and activates Ca-dependent genes. Changes in Ca level lead to ROS production and an increase in gene expression. Cadmium activates cellular protein kinases, leading to increased phosphorylation of transcription factors and an increase in certain gene expression [149]. Additionally, cadmium exposure inhibits DNA-methyltransferase-1 (DNMT1) and decreases DNA methylation, which can be associated with cell transformation and hyperproliferation. Cadmium exposure inhibits DNA repair and damages the genome, leading to potential cell transformation. It interferes with repair processes and inhibits DNA repair genes expression, transcription factor activity, and protein function. Additionally, cadmium can replace zinc in proteins, leading to nonfunctional enzymes. Zinc supplementation can help correct some of the DNA damage [149].

**Figure 7:**
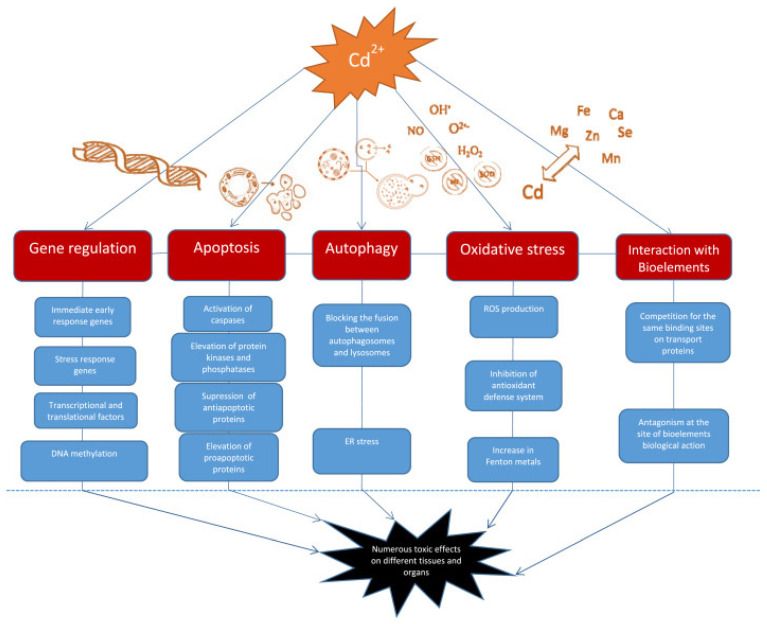
General mechanisms and specific molecular pathways of Cd toxicity. The figure shows general mechanisms (gene regulation, apoptosis, autophagy, oxidative stress, and interaction with bioelements) along with the specific molecular pathways of Cd toxicity [149].

Autophagy, a process of self-degradation that plays a crucial role in eliminating proteins and clearing damaged organelles, is increasingly acknowledged to be involved in Cd toxicity. Cd can function as both a protector and a promoter of cell death [150]. The conflicting impact on cell fate is determined by the appropriate level of autophagy needed to sustain cell survival. Cd exposure disrupts normal cellular autophagy, and both excessive autophagy and its absence can lead to cell death [148]. The effects of Cd on the autophagy process are observed as either stimulation or disruption, likely based on gene expression [148]. Autophagy following Cd exposure appears to either suppress or trigger apoptosis, as the increased accumulation of ROS can activate both autophagy and apoptosis. Moreover, Cd-induced elevation of intracellular Ca leads to ROS induction, initiating cell apoptosis due to the interaction between Ca signaling and ROS in normal and pathological conditions (Figure 7) [149].

Cd triggers cell death by modifying the behavior of protein kinases such as mitogen-activated protein kinase (MAPK). Cd enhances the activity of p38-mitogen-activated protein kinase (p38 MAPK), leading to increased expression of genes related to inflammation and cell death, ultimately resulting in tissue necrosis and kidney damage in rats. Furthermore, recent findings show that Cd prompts cell death in TM3 Leydig cells by generating reactive oxygen species (ROS) and promoting phosphorylation via the JNK pathway. Consequently, this causes a reduction in the levels of the anti-cell death protein Bcl-2, followed by the activation of caspase-3 and cell demise [149].

Exposure to Cd impacts the functioning of glutamate, acetylcholine, GABA (gamma-aminobutyric acid), and dopamine neurotransmitter receptors in the brain. N-methyl-D-aspartate receptor (NMDAR) voltage-dependent calcium channels facilitate neuronal uptake of Cd, which leads to increased Cd influx following stimulation with glutamate or N-methyl-D-aspartate (NMDA) and glycine. Additionally, Cd interacts with muscarinic acetylcholine receptors, leading to cell death in primary cholinergic neurons from the basal forebrain by suppressing the muscarinic receptor M1. A subsequent study by the same authors demonstrated that oxidative stress caused Cd-induced muscarinic receptor disruption [150].

Early research suggests that Cd inhibits the neuronal GABA_A_ receptor channel complex through a binding site different from the recognition sites for GABA and other drugs. Moreover, exposure to Cd alters the expression of GABA_A_ receptors in animal studies. Specifically, altered protein expression levels of GABA_A_Rα5 and GABA_A_Rδ were observed in the hippocampus of mice offspring following Cd exposure during pregnancy and lactation, indicating that GABA_A_Rα5 is more susceptible to environmental pollutants during puberty and young adulthood. Conversely, GABA_A_Rδ may reflect the accumulation of environmental contaminants in adulthood [150].

**Figure 8:**
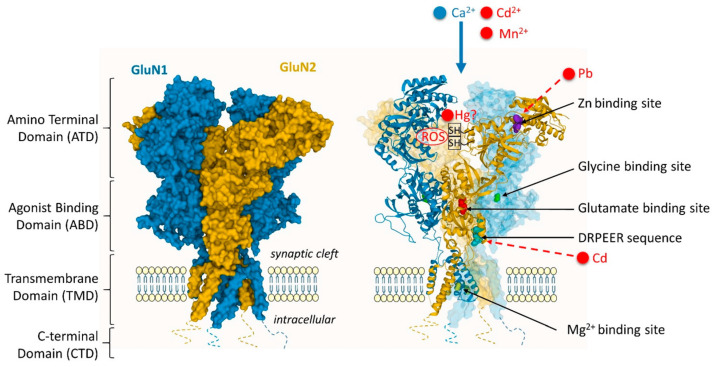
Interaction sites of environmental metal toxicants with the NMDA receptor are as follows: Cd^2+^ facilitates neuronal uptake after receptor stimulation. Similarly, Mn^2+^ can enter the plasma membrane through the NMDAR. Cd can directly attach to the DRPEER sequence in the extracellular domain (in the agonist binding domain (ABD)/transmembrane domain (TMD) linker) of the GluN1 subunit, inhibiting NMDA-mediated current. Pb competes with zinc for the zinc-binding site of the GluN2 subunit, thereby affecting receptor function. As for Hg, there are indications of interactions with cysteine -SH groups that regulate NMDAR activity, although this suggestion still requires experimental evidence. In most instances, metals may induce ROS, which can interact with the –SH groups of the NMDAR [150,151].

Cd-induced neurotoxicity causes impairments in movement due to Cd’s specific impact on DA receptors. Cd exposure reduces the production of mRNA and proteins associated with dopamine (DA)-D2 receptors in the stratum of rat brains. In contrast, the levels of expression for DA-D1 receptors remain unchanged. Additionally, experiments using molecular docking have shown that Cd may directly attach itself to the competitive site of dopamine on DA-D2 receptors (Figure 8) [150].

CdCl_2_ leads to the disassembly of the cytoskeleton in various cultured neuronal cells, affecting both the actin and microtubule networks. In primary rat cortical neurons, Cd causes the destruction of microtubules and reduces acetylated tubulin levels. Furthermore, Cd down-regulates the gene expression of microtubule dynamics and microtubule motor-based proteins in a neuronal human cellular model [150,152].

**Figure 9:**
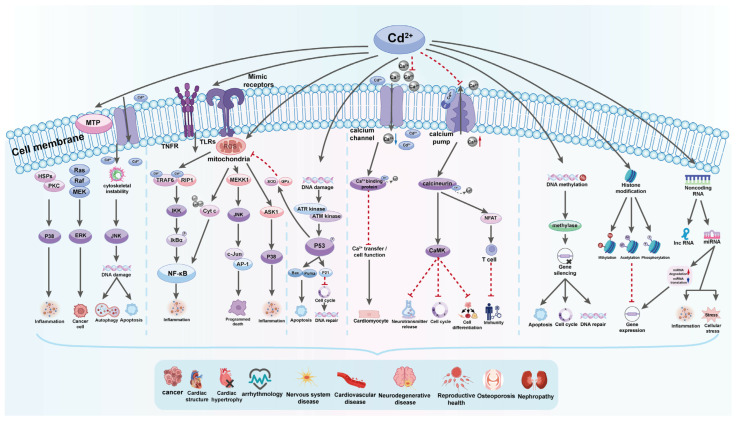
Cd’s effect on MAPK pathways disrupts cellular signaling and health. The figure demonstrates how Cd^2+^ disrupts the MAPK signaling pathways, highlighting direct and indirect impacts on cellular function. This leads to abnormal cell responses and an increased risk of disease. Cd’s role in activating NF-κB signaling is a pathway to increased inflammation. Cd^2+^ activates the NF-κB pathway and interacts with ROS, receptors, and kinases, resulting in heightened inflammation and potential chronic conditions. Cd^2+^ also affects the p53 pathway, which leads to DNA damage responses, including apoptosis and cell-cycle control. The role of p53 is in protecting cellular health. Cd^2+^ disrupts Ca^2+^ homeostasis by mimicking and interfering with Ca^2+^ in cells. The figure depicts the effects of Cd on calcium channels and pumps and the subsequent risks to cell function and activity. The figure also demonstrates the pathways through which Cd^2+^ influences epigenetic processes, impacting DNA methylation, histone modification, and non-coding RNA activity. These changes lead to significant alterations in gene expression, affecting cellular functions and contributing to disease progression, particularly in cancer and other severe health conditions [114].

The toxic effects of Cd on human health are widespread and are caused by various biochemical and molecular mechanisms. The main ways in which Cd causes harm include inducing oxidative stress, disrupting Ca^2+^ signaling, interfering with cellular signaling pathways, and making epigenetic modifications. Cd interacts with cellular components such as mitochondria and DNA and causes extensive damage at both cellular and tissue levels [114]. Cd induces oxidative stress, which is a crucial mechanism behind its toxicity, and thus disrupts the balance between oxidants and antioxidants, leading to cellular damage and apoptosis. Furthermore, Cd negatively impacts signaling pathways such as mitogen-activated protein kinase (MAPK), nuclear factor kappa-light-chain-enhancer of activated B cells (NF-κB), and tumor protein 53 (p53) pathways. Cd’s interference with these pathways contributes to pathological conditions and carcinogenesis. The epigenetic effects of Cd include DNA methylation and histone modifications, and it causes long-term impact on gene expression and disease manifestation (Figure 9) [114].

## 5. Methods for Cd Removal

### 5.1. From the Environment

#### 5.1.1. Chemical Precipitation

Chemical precipitation is the most efficient and widely used technology for removing Cd^2+^ from wastewater because it is simple, inexpensive, and easy to apply. In this procedure, substances react with Cd^2+^ to produce insoluble precipitates [153]. Precipitants such as hydroxides and sulfides are often employed to precipitate Cd^2+^, resulting in insoluble Cd(OH)_2_ and CdS residues, respectively. Simple sedimentation or filtration procedures could remove insoluble precipitates from water before they are released into the environment or reused [50].

#### 5.1.2. Adsorption

Adsorption is another excellent way to remove Cd^2+^ from wastewater. Physiological interactions transfer metal ions from the aqueous to the solid phase [154]. Mineral clay, mineral ores, agricultural wastes, synthetic polymers, and industrial leftovers are all examples of low-cost absorbent materials [41].

#### 5.1.3. Ion Exchange

This approach uses the ion exchange principle to adsorb Cd^2+^ on solid surfaces. Metal ions in solution are replaced by ions bonded to the surface of an insoluble matrix, commonly known as an ion exchanger, and covalently bound to negative or positive functional groups. As a result, these functional groups are connected to oppositely charged ions, which are then replaced by the same charged ions found in the solution due to their higher affinity for the functional groups. Many inexpensive materials, such as waste iron, fly ash, resins, zeolites, and silicate minerals, have been widely used as ion exchangers [50]. pH and temperature are the most important parameters influencing ion exchangers’ uptake of metal ions.

#### 5.1.4. Membrane Filtration

Membrane filtration is a practical approach for removing hazardous metal ions from polluted water. Several methods use membranes to remove metal ions from effluent, including nanofiltration, ultrafiltration, electrodialysis, and reverse osmosis. Generally, metal-containing wastewater is passed over membranes with varying pore diameters depending on the nature and kind of metal ions to be extracted using selective pressure or electric current (for example, in electrodialysis). The membrane’s pore size is smaller than the metal ion, preventing metal ions from passing through and decontaminating the effluent.

All conventional treatment methods have disadvantages, such as waste byproducts, sludge production, membrane fouling, low selectivity and efficiency, and high capital and operating costs [155]. Thus, using living and dead microbial biomass to remove heavy metal ions from the environment has gained popularity due to its benefits, such as cost-effectiveness, efficiency, and environmental friendliness [156].

### 5.2. From the Human Body

Nano selenium-enriched probiotics have been investigated recently for their potential in addressing Cd liver toxicity [157]. The presence of Cd and other heavy metals like Pb in the gut and food-derived microbes can actively or passively affect the bioavailability of these toxins within the gut [158]. Probiotic treatment can alleviate Cd-induced cytotoxicity, reduce oxidative stress and inflammation, restore tight-junction integrity, and lower gut permeability in intestinal epithelial cells and animals. This safeguarding of the gut barrier leads to higher levels of fecal Cd and reduced Cd accumulation in mouse tissues, indicating probiotics’ ability to inhibit intestinal Cd absorption [159]. Capriglione et al. studied the impact of CdCl_2_ on immortalized, non-tumorigenic thyroid cells Nthy-ori-3-1 and the protective influence of quercetin against CdCl_2_-induced damage [160]. Nutritional trace metals such as Zn and Se have the ability to alleviate Cd-induced mitochondrial toxicity. Furthermore, Se supplementation can reduce Cd-induced oxidative stress and the mitochondrial apoptosis pathway [161]. A study reported by Smereczanski et al. found that an extract from *Aronia melanocarpa* L. berries has a protective effect against Cd in a rat model. The co-administration of that extract significantly reduces the harmful effect of Cd-induced kidney damage [162]. Resveratrol has also been found to have a protective effect against hepatotoxicity, according to Al-Baqami et al. [163]. Magnesium chloride was reported to diminish Cd toxicity via drinking water with a concentration of 500 mg Mg/L [164]. Asperuloside, an iridoid monoterpenoid glycoside found in many medicinal plants, is described as a substance attenuating Cd-induced toxicity [165].

## 6. Cd Resistance Mechanisms

Some microbes, such as bacteria, absorb heavy metals such as Cd^2+^ and necessary metal ions, helping to eliminate hazardous metal ions from the aquatic environment [158]. Heavy metal ions generate ROS and thus disorder bacterial metabolism by damaging DNA, RNA, and proteins. Hyperaccumulation of Cd^2+^ may cause bacterial respiratory proteins to disorganize, disrupting the physiological functioning of the cell. To battle the fatal effects of heavy metals, bacteria have evolved various metal resistance methods, including biosorption, efflux transport, intracellular and extracellular sequestration, transformation, and physiological adaptations [166].

Organisms have mechanisms to detoxify Cd, including chelation, compartmentalization, and efflux. In yeast *S. cerevisiae*, yeast cadmium factor 1 (YCF1) provides resistance to Cd by sequestering glutathione-conjugated cadmium, bis(glutathionato)Cd [167]. Efflux and sequestration of heavy metals via metallothioneins and other molecules containing thiol groups are well-known metal resistance mechanisms in bacteria against Cd^2+^ and are briefly described below [50].

ABCC proteins in humans are linked to the detoxification of chemicals, their distribution, and regular cell functions. In Saccharomyces cerevisiae, the ABCC protein ScYCF1 has the ability to provide resistance to Cd. In plant systems, ABCCs are known to be involved in detoxifying and sequestrating potentially harmful elements, as well as transporting chlorophyll breakdown products and regulating ion channels [168]. For instance, in *Arabidopsis*, AtABCC1 facilitates the export of glutathione-S (GS) conjugates from the cytosol. Furthermore, the AtABCC1 and AtABCC2 genes have been associated with the vascular sequestration of phytochelatin (PC)-Cd(II) and PC-Hg(II), while the AtABCC3 gene encodes a transporter for PC-Cd complexes. The wheat ABCC protein, TaABCC13, is crucial for the glutathione-mediated detoxification process, and OsABCC1 in rice lowers As levels in grains by sequestering As in the vacuoles of phloem companion cells within diffuse vascular bundles. These observations underscore the importance of ABCC members in understanding the transport and detoxification of potentially toxic elements [168]

### 6.1. Biosorption

Bacteria and yeasts have the intrinsic ability to bioabsorb metal ions due to their unique cell envelope, which limits cellular intake of harmful metal ions to preserve homeostasis [169]. When dealing with microbial organisms such as yeast or bacteria, the cell wall is the first structure interacting with metal ions. Several studies have indicated the existence of critical functional groups on biomass/biomaterial surfaces, such as hydroxyl, thiol, carboxyl, and amino groups, which play a crucial role in metal ion biosorption [170]. Nonetheless, the specific process of Cd^2+^ biosorption is unknown and varies depending on factors such as biomass type, heavy metal properties, co-metal ion presence, pH, and medium temperature. The most influential factor is the composition of biomaterials’ surfaces [50].

Understanding biosorption’s mechanism requires a better understanding of the cell surface’s structure and chemical makeup. Gram-negative bacteria’s cell wall comprises peptidoglycan, phospholipids, and lipopolysaccharides. The lipopolysaccharides have a negative charge, contributing to the cell wall’s anionic character. The cell wall of Gram-positive bacteria contains about equal amounts of peptidoglycan and teichoic acids (TAs). While both carry an anionic charge at neutral pH, TAs (anionic, phosphate-based linear polymers) are critical in keeping a net negative charge on the bacterial surface [50].

### 6.2. Efflux Transport Systems

Heavy metals enter microbial cells via the critical metal ion absorption pathways earlier described in this review. Cd uses manganese and magnesium transport systems in Gram-positive and Gram-negative bacteria. Cd hyperaccumulation causes the cell to produce efflux systems for its removal to maintain homeostasis. The efflux systems may be chromosomal or plasmid controlled. Three distinct efflux systems, notably resistance nodulation cell division (RND), P-type ATPases, and cation diffusion facilitator (CDF), have been discovered in bacteria to remove heavy metal divalent cations such as Cd^2+^ from the cells [50].

## 7. Conclusions

Cd’s toxic effects, environmental impact, sources, health effects, and biological impacts make it a pressing issue. This review highlights its toxic nature, widespread pollution, entry into living organisms’ systems, including the human body, through ingestion, inhalation, and permeation, and its accumulation in organs, leading to severe health issues. Cd exposure leads to extensive environmental and health harm. Cd is highly toxic and can be absorbed by plants and crops from the soil, then enters the animal body, leading to potential exposure for humans through the food chain. It accumulates in various organs, particularly the kidneys and liver, and is known to cause severe health problems, including renal dysfunction, bone diseases, cardiovascular problems, and many others. On a cellular level, Cd disrupts numerous biological processes, inducing oxidative stress generation and DNA damage, and is classified as a carcinogen by the International Agency for Research on Cancer (IARC). Preventing the use of products containing Cd is essential to minimize its adverse effects on humans and other living beings. The current Cd usage trend will lead to more severe consequences if it continues. To avoid Cd toxicity, restoring Cd-contaminated sites and appropriately disposing of materials containing Cd is essential.

## Figures and Tables

**Table 1:** Physical and chemical properties of Cd [4].

Atomic number	48
Atomic weight	112.41 u
Atomic radius	155 pm
Electronic configuration	[Kr]4d^10^5s^2^
Melting point	321.07 °C
Boiling point	767.3 °C
Density at 20 °C	8.65 g/cm^3^
Reduction potential Cd^2+^ + 2e^−^ → Cd(s)	−0.40 E°
Heat of fusion	6.21 kJ/mol
Heat of vaporization	99.6 kJ/mol
Electronegativity (Pauling scale)	1.69
First ionization energy	867.8 kJ/mol
Second ionization energy	1631.4 kJ/mol

**Table 2:** Cd^2+^ removal (%)/uptake (mM/g) potential in some bacterial and yeast strains [50].

Organism	Cd^2+^ Resistance (mM)	Cd^2+^ Removal (%) Uptake (mM/g)
*Klebsiella pneumoniae*	13.3	57.4
*Escherichia coli* P4	10.6	56
*Salmonella enterica* 43C	13.3	22
*Bacillus* sp.	–	50
*Rhodobacter sphaeroides*	–	30.7
*Microbacterium oxydans* CM3	–	43
*Candida tropicalis*	25.0	92
*Pichia hampshirensis* 4Aer	24.0	28
*Candida tropicalis* 3Aer	25.1	31
*Trametes versicolor*	5.0	0.300
*Trichosporon ashii*	10	78
*Pichia kudriavzevii*	15	61

**Table 3:** Plant species show varying responses to Cd-induced DNA damage. This damage includes DNA strand breaks, chromosomal aberrations, and micronuclei formation. Cd also impacts the expression of DNA repair genes and causes alterations in amplified fragment length polymorphism (AFLP), inter-simple sequence repeat (ISSR), random amplified polymorphic DNA (RAPD), sequence-related amplified polymorphism (SRAP), and simple sequence repeat (SSR) profiles, leading to a reduction in genomic template stability (GTS). The symbols ↑ and ↓ indicate increases and decreases, respectively [64].

Species	Organ	Cd Concentration	Exposure Duration	Effect
*Allium cepa*	Root tip	50–200 µM	2 h + 24 h recovery	Micronucleus formation Chromosomal aberrations
	Root tip	25 µM	48 h	
	Root tip	25 µM	48 h	% tail DNA ↑
*Arabidopsis thaliana*	Root tip	0.125–2.5 mg L^−1^	5 d	Altered expression DNA repair genes
	Root	1.25–4 mg L^−1^	5 d	Altered RAPD profile Altered expression DNA repair genes
	Leaf	0.5–5 mg L^−1^	16 d	Altered AFLP profile
	Leaf	0.25–8 mg L^−1^	15 d	Microsatellite instability Altered RAPD profile
	Leaf	5 µM	72 h	Altered expression DNA repair genes
*Brassica chinensis*	Leaf	15–120 mg kg^−1^ soil	30 d	Altered RAPD profile
*Brassica oleracea*	Root	2.5–20 mg kg^−1^ soil	3–56 d	Altered % tail intensity
*Capsicum annuum*	Root tip	20–100 ppm	24 h	Chromosomal aberrations
	Leaf	20–100 ppm	24 h	Altered RAPD profile
*Hordeum vulgare*	Root tip	75–225 µM	7 d	Altered RAPD profile (GTS ↓)
	Leaf	5 µM	15 d	DNA damage ↑
*Ipomoea aquatica*	Entire seedling	15–120 mg kg^−1^ soil	21 d	Altered RAPD profile (GTS ↓)
*Lactuca sativa*	Root tip	25 µM	48 h	Chromosomal aberrations Micronucleus formation % DNA damage ↑
*Lathyrus sativus*	Root tip	5–50 µM	3–7 d	Chromosomal aberrations Micronucleus formation
*Leucaena leucocephala*	Leaf	50 mg L^−1^	15 d	Altered RAPD profile
*Nicotiana tabacum*	Root and leaf	10–15 µM	7 d	% tail DNA ↑
*Oryza sativa*	Root tip	50–200 µM	48–96 h	Altered SRAP profile (GTS ↓)
*Sphagnum palustre*	Shoot	0.1–10 µM	24–48 h	Altered ISSR profile (GTS ↓)

**Table 4:** An analysis of the effects of Cd on cell cycle-related parameters organized by plant species. Exposure to Cd decreases the mitotic index (i.e., the ratio of cells undergoing mitosis to the total number of cells) and changes in nuclear ploidy levels. It influences the expression of cell cycle-related genes in various plant species. The symbols ↑ and ↓ represent increases and decreases, respectively [64].

Species	Organ	Cd Concentration	Exposure Duration	Effect
*Allium cepa*	Root tip	50–200 µM	2 h + 24 h recovery	Mitotic index ↓
	Root tip	25 µM	48 h	Mitotic index ↓
*Arabidopsis thaliana*	Root tip	0.125–2.5 mg L^−1^	5 d	2C ↓, 4C ↑, 8C ↑ Altered cell cycle phase distribution Altered expression cell cycle-related genes
	Root	1.25–4 mg L^−1^	5 d	2C ↓, 4C ↑ Altered expression of cell cycle-related genes
	Leaf	5 µM	3–12 d	Endoreduplication factor ↓ Epidermal cell number and cell surface area ↓ Altered expression of cell-cycle related genes
*Capsicum annuum*	Root tip	20–100 ppm	24 h	Mitotic index ↓
*Lactuca sativa*	Root tip	25 µM	48 h	Mitotic index ↓
*Lathyrus sativus*	Root tip	5–50 µM	3–7 d	Mitotic index ↓
*Oryza sativa*	Root	200 µM	7 d	Cortex cell length in the elongation zone ↓ Cortex cell number in the elongation zone ↓ Altered expression of cell cycle-related genes
*Sorghum bicolor*	Root tip	50–200 µM	5 d	Inhibition of S phase progression

## Data Availability

The data used during the current study are available from the corresponding author upon request.
